# Editorial: Mental health promotion during COVID-19: applications from self-care resources, lifestyles, and environments

**DOI:** 10.3389/fpsyg.2023.1213288

**Published:** 2023-06-01

**Authors:** Martín Martínez, Elkin O. Luis, Francisco Ceric, Elena Bermejo-Martins

**Affiliations:** ^1^Methods and Research in Affective and Cognitive Psychology, School of Education and Psychology, University of Navarra, Pamplona, Spain; ^2^Psychological Processes in Education and Health Group, School of Education and Psychology, University of Navarra, Pamplona, Spain; ^3^Affective Neuroscience Laboratory, Faculty of Psychology, Universidad del Desarrollo, Santiago, Chile; ^4^Department of Community Nursing and Midwifery, School of Nursing, University of Navarra, Pamplona, Spain

**Keywords:** COVID-19, mental health crisis, self-care, health psychological wellbeing, pandemic (COVID19)

The COVID-19 pandemic has had profound and wide-ranging effects beyond its medical implications, causing significant sociological, psychological, and economic consequences on a global scale. This pandemic posed a significant social challenge, and it has also provided us with invaluable lessons learned from facing this unprecedented situation. Specifically, recognizing the significance of self-care as a psychological resource in managing the pandemic mental health consequences, which has become increasingly crucial, and will continue to be critical in future public health crises.

The WHO defines self-care as: “the ability of individuals, families and communities to promote health, prevent disease and maintain health, and to cope with illness and disability with or without the support of a healthcare provider” (World Health Organization, 2009, p. 17). Self-care is not solely for personal benefit, as it also has the potential to impact the social and political environments. These environments can, in turn, enable self-care for individuals who grow up and interact within those spaces, leading to a ripple effect of potential wellbeing. This fact highlights the importance of individuals that engage in self-care or health-protection activities as citizens, as their actions can have a collective impact and influence others.

In light of these considerations, we announced the launch of our Research Topic on October 22nd, 2021. This editorial piece highlights the contributions to the impact of self-care practices in promoting mental wellbeing and mitigating the mental health problems caused by the COVID-19 pandemic in different populations. A total of 14 articles were finally published, comprising 13 research articles and one opinion piece, authored by 87 scholars from eight different countries (Figure 1).

**Figure 1:**
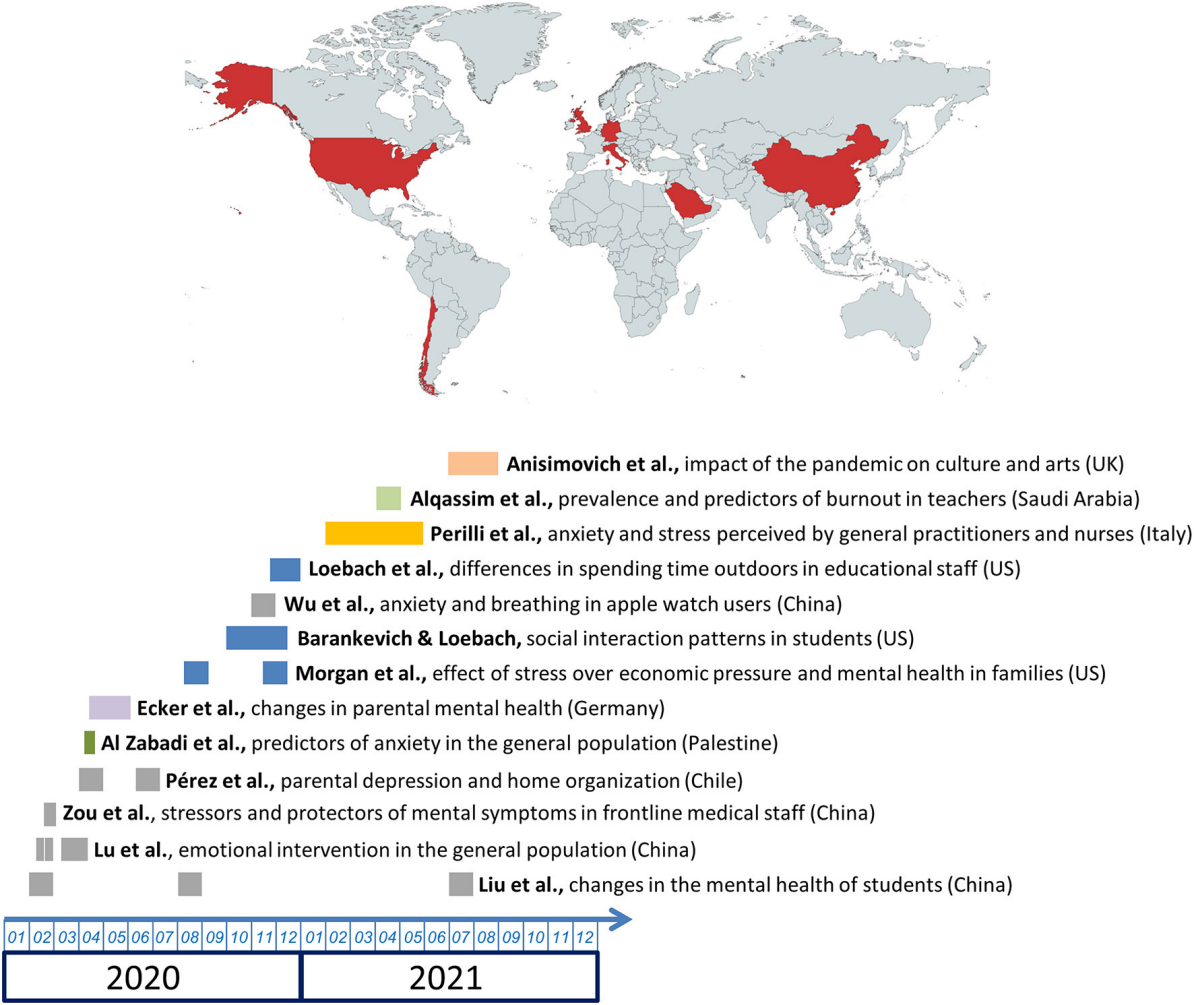
Chronology of data collection and general objectives for research articles published on the current Research Topic, along with a worldwide map depicting the countries where the works were conducted (in red).

The manuscripts were featured in the section of Health Psychology and their primary focus was on one of these three key themes.

## 1. Exploring the impact of social determinants of health on the ability of the general population to engage in self-care

Since the beginning of the pandemic, lockdown measures have caused a remarkable negative emotional impact on the general population, as noticed in the increase in negative mental symptoms such as stress, anxiety, or depression. Al Zabadi et al. investigated the prevalence and predictors of stress and anxiety among Palestinian young adults at the onset of the pandemic. Their findings indicated that those with low socioeconomic status and inadequate access to food were at greater risk for elevated levels of stress and anxiety. In contrast, Wu et al. proposed a novel approach to anxiety management through guided respiration practice, employing smartwatches, which they tested on a Chinese population. Their results demonstrated the potential efficacy of this intervention in controlling anxiety levels.

Lu et al. conducted a study on emotional changes in the Chinese population during the first months after the COVID-19 outbreak, utilizing an online group psychological intervention model. Their findings indicated that people's emotions quickly shifted from stress, anxiety, and isolation to hope for a return to normalcy. The group psychological crisis intervention model proved to be effective in regulating negative emotions and provided members with a safe space for communication. However, the resumption of in-person events has been slow and gradual, necessitating measures such as control of capacity of closed-spaces and prohibiting indoor activities. Anisimovich et al. investigated the impact of the pandemic on cultural and arts engagement patterns, with a focus on the experiences of the audience in Liverpool (UK). The study highlighted the pandemic's influence on the ways people engage with the arts and culture, which were seen as helpful in rebuilding personal resilience and confidence. These findings underscore the importance of providing support for individuals and communities to cope with the emotional and social impacts of the pandemic.

## 2. Risk factors and resilience at the family level

This pandemic has also changed the organization of families at home. A study conducted in Germany during the early phase of the pandemic by Ecker et al. found that parents were not necessarily at risk due to additional burdens, but instead they had better prospects for coping than those without children. In a longitudinal study carried out on Chilean parents, Pérez et al. reported an increase in depression and home chaos with respect pre-pandemic levels, which was moderated by factors such as financial stability, personality, and recent major stressful events. Additionally, research conducted among parents in Texas (USA) by Morgan et al. found that economic pressure led to worsened mental health, with stress mediating this pathway. Moreover, approach coping strategies were found to be more effective in moderating the association between stress and later anxiety symptoms compared to avoidant coping strategies. Sánchez-Hernández et al. suggested the implementation of interventions aiming at the promotion of resilience and emotional self-care to mitigate the impact of pandemics on families and healthcare professionals.

## 3. Demands and resources in two main professional sectors at risk: healthcare and education

Regarding the healthcare sector, the outbreak increased work demands for healthcare professionals, which had a particular impact on their mental health. Zou et al. explored the role of perceived stress and social support in explaining the relationship between three negative symptoms (e.g., occupational stressors and anxiety, depression, and insomnia) among the frontline medical staff at the beginning of the pandemic in Wenzhou (China). They reported that occupational stressors were related to all negative symptoms, with perceived stress mediating, and social support moderating these associations. Perilli et al. evaluated perceived anxiety and stress by general practitioners and by hospital ward nurses in the district of ASL1 Avezzano-Sulmona-L'Aquila (Italy) during acute phase of the pandemic. They found higher levels of anxiety in general practitioners than among nurses, which were associated with increases in emotional distress. In addition, anxiety increases were coped with by enlarging the demand for social support in general practitioners. This coping strategy correlated with emotional distress and when enhanced, it corresponded to avoidance of the problem.

The pandemic also interrupted education-learning processes, which added more stressors to students, teachers, and education staff since they had to change their working styles. Alqassim et al. examined the prevalence and the associated factors of burnout among schoolteachers in the Jazan Region (Saudi Arabia) in April 2021. Authors indicate that most teachers showed burnout symptoms, which were related to taking psychotropic medications, absenteeism, lack of job satisfaction, and school change. On the other hand, being an expert and having the ability to adapt to technology proved to reduce burnout symptoms effectively. Loebach et al. explored whether education staff from the state of New York (USA) spent more time outdoors during the pandemic as compared to pre-pandemic times. The majority of participants reported spending more time in natural outdoor environments since the pandemic had emerged, particularly in its early stages. The relative accessibility of those environments impacted the behavior of the participants, In terms of the number of occasions being outdoors and the number of natural outdoor environments visited. Liu et al. investigated the dynamic changes in the mental health status of Chinese university students in Xuzhou (province of Jiangsu) since the outbreak of the pandemic and after 1 year. They reported that the degree of recognition of the pandemic was an important factor that significantly affected the psychological state of college students, and that effective control of the environment slightly improved the behavior and mental state of the students. Similarly, Barankevich and Loebach explored the social interaction patterns of university students in northeastern USA during the outbreak of COVID-19. They suggested that students had spent significantly less time interacting with non-roommates in person during the pandemic, and more time in voice and video calls, and that meaningfulness had been higher for interactions with family or friends.

In summary, the aforementioned studies provide robust evidence about the importance of considering the social determinants of mental health to promote mental wellbeing, and the central role self-care plays. It has been highlighted that self-care can positively influence health attitudes and behaviors in the population. Promoting mental health with a central focus on self-care enhances the quality of life, alleviates clinical symptoms, and increases awareness and use of available individual and collective health resources. Additionally, evidence supports the effectiveness of collective and individual actions on self-care, as they can favor healthy environments and lifestyles, and facilitate citizen reflection on self-management practices from a context-based perspective. Likewise, self-management practices can guide the implementation of targeted actions aimed at reducing the most prevalent health risks in the community. Thus, future public health recommendations should carefully consider the role of community-based decision-making actions that involve actively citizens to empower them to adopt and influence self-care behaviors, emphasizing problem-solving and collaborative development with health, social and political sectors.

## Author contributions

EL, MM, and EB-M were responsible for the manuscript design. All authors contributed to this work and approved the final manuscript.
